# The relationship between parental self-efficacy, health-related quality of life, stress and educational level on adolescents’ self-efficacy: a cross-sectional study

**DOI:** 10.1186/s41043-025-01163-z

**Published:** 2025-12-29

**Authors:** Erik Grasaas, Gudrun Rohde, Hilde Timenes Mikkelsen, Sølvi Helseth, Milada Hagen, Siv Skarstein, Kristin Haraldstad

**Affiliations:** 1 Teacher Education Unit, University in Agder, Postbox 422, 4604 Kristiansand, Norway; 2 Department of Health and Nursing Science, Faculty of Health and Sport Sciences, University in Agder, Kristiansand, Norway; 3 Department of Nursing and Health Promotion, Faculty of Health Sciences, Akershus University College of Applied Sciences, Oslo, Norway; 4 Department of Clinical Research, Sorlandet Hospital, Kristiansand, Norway

**Keywords:** Adolescents, Self-efficacy, Stress, Parents, Health-related quality of life

## Abstract

**Background:**

Self-efficacy is a well-known concept often referred to as the belief in the ability to perform in different situations. The concept of self-efficacy is particularly relevant during adolescence, as it serves as a self-regulatory mechanism through which adolescents can be motivated to change their behavior by significant others, such as parents. However, there is little research evidence on how parental factors are associated with adolescents’ self-efficacy. Hence, this current paper aimed to describe parental self-efficacy, health-related quality of life (HRQOL), stress, and educational level and explore associations between the participating parent’s self-efficacy, HRQOL, stress, and educational level on adolescents’ self-efficacy stratified by gender.

**Methods:**

A cross-sectional study was performed among 508 Norwegian adolescent-parent dyads. Adolescents were 13–15 years old and completed an electronic survey during school hours with teacher and researcher present, whereas the participating parent completed the survey at home. The Survey comprised of a test battery of questionnaires, including sociodemographic data, self-efficacy for both adolescents and parents, and the parental factors: stress, HRQOL, and educational level. Separate multivariable regressions were conducted for the participating parent using STATA software.

**Results:**

Descriptive analyses revealed a self-efficacy score of (mean/standard deviation (SD), 32.9 (4.0) vs. 34.1 (4.3)), HRQOL (mean/SD, PCS 51.1 (9.5) vs. 53.3 (7.0), MCS 51.9 (8.2) vs. 54.1 (7.2)) and stress (mean/SD, 0.28 (0.24) vs. 0.25 (0.15) among mothers and fathers respectively. Half of the mothers (506%) and 46.1% of the fathers reported ≥ 16 years of education. Multivariable regressions revealed all nonsignificant associations of all parental study variables on adolescents’ self-efficacy (all *p* > 0.05).

**Conclusions:**

In this study, the parental factors examined were not associated with adolescents’ self-efficacy. These findings highlight the need for further observational and longitudinal studies with larger samples to better understand how parental factors influence adolescents’ self-efficacy.

## Background

Adolescence is a demanding period of independence with the arise of numerous new challenges, herein adolescents are exposed to new social situations in combination with physical and psychological development [1, 2]. The period entails development of the social brain, where the network of brain regions expand and influence the social cognition and behavior [3]. During the last decades, our understanding of social cognition and human behavior have been explained by the social cognitive theory developed by Albert Bandura [4]. This theory suggests that adolescents’ performance or behavior is influenced by their beliefs and support by peers and parents [5]. According to Bandura, self-efficacy is the most suitable approach to influence beliefs and is defined as “how well one can execute courses of action required to deal with prospective situations” [6].

Self-efficacy is a well-known concept, which is often referred to as the belief in the ability to perform in different situations [7] and have been included in extensive research evidence, even incorporated in health behaviour change models such as the Health Belief Model and Theory of Planned Behavior due to its relevance [8, 9]. Hence, self-efficacy has been identified as an important determinant of both present and future health behaviour [10]. The concept of self-efficacy is especially interesting in adolescence due to the self-regulatory mechanism by which it is possible to change as a result of being motivated by the significant others, presumably parents, peers, education or goal-setting [11, 12]. Bandura argues that self-efficacy will, directly or indirectly influence our behaviour through impacting goals, outcome expectations and perception of sociostructural factors [13].

Self-efficacy is considered a major predictor for healthy behavior [14–16] and is positively associated with several beneficial health outcomes in adolescence, such as lower stress, less aggression, smoking and misuse of alcohol, higher health-related quality of life (HRQOL), and better school performance [17–24]. Several Norwegian studies have reported positive associations between self-efficacy in Norwegian adolescents and HRQOL [25–27]. HRQOL is conceptualized as multidimensional, including physical, mental, and social well-being and serves as a relevant framework due to the positive focus on the subjective perception of resources [28]. Moreover, self-efficacy is reported as a key variable in the appraisal process and especially linked to stress outcomes [29]. Research evidence has shown that possessing high levels of self-efficacy decreases a person’s risk of experiencing stress [29–32]. Stress is defined by Lazarus and Folkman, as “a relationship between the person and their environment that is appraised by the person as taxing or exceeding their resources and as endangering their well-being” [33].

As self-efficacy is linked to several positive HRQOL outcomes, possible approaches for increasing self-efficacy should be highlighted. Close to a half century ago, Bandura argued that self-efficacy is affected by the following components [5]: (I) Performance accomplishments (personal experience), (II) Vicarious experiences (observations of others), (III) Verbal persuasion (encouragement), and (IV) A person’s physiological state (physiological reactions). Interestingly, when accumulated the relevant research evidence decades later in a systematic review with meta-analysis by Ashford and colleagues, investigating the best way to change self-efficacy to promote a healthy lifestyle, their findings were close to be in accordance with the proposed components of Bandura. Findings revealed that feedback by comparing performance, and feedback on the participant’s past performances and vicarious experience were the most effective way to promote self-efficacy [14]. Hence, as children and adolescents are learning through their observation of others, it is a logical assumption that parents and caregivers might play a key role as both role models and by providing constructive feedback. It is likely that several characteristics and mechanisms in parents that may affect adolescents’ self-efficacy, including both supportive and limiting factors. Parental health status and high parental empowerment, may increase their ability to cope with everyday parental-related demands [34], however mechanisms related to parenting practices would provide a more comprehensive understanding of the context, as previous research have suggested strong relations between parental practices and adolescents well-being [35], which is closely related to self-efficacy among Norwegian adolescents [25–27].

Moreover, stress in parents has been shown to negatively affect parenting practices and thereby could influence both their own and adolescents’ self-efficacy. According to Senn and colleagues, parental stress mediates the effects of parental risk factors on dysfunctional parenting in first time parents [36], unveiling parental stress as a key variable related to adolescents’ self-efficacy. Since parental stress may shape how adolescents perceive their own ability to cope with challenges, investigating the influence of parental factors on adolescents’ self-efficacy is particularly important.

In self-efficacy as a positive youth development construct: a conceptual review, by Tsang et al., the authors acknowledged that most parents put a very high priority on supporting their adolescents to achieve academically [7] and has earlier reported that home-schooling is useful for adolescents’ development [37]. However, in the review by Klassen et al., on self-efficacy in educational settings [38], only 2% of the total of 244 articles included parents. Moreover, within families, there are differences between how mothers and fathers interact with their adolescents. Research has shown that parents’ self-efficacy beliefs and parents’ gender are related to their children’s reading achievement, especially favoring mothers’ impact [39].

As adolescents may learn from observation of parents, it is interesting to explore how parental educational level is associated with adolescents’ self-efficacy, as well-educated and resourceful parents may help the adolescents by supporting homework-related school assignments [37]. Moreover, higher parental self-efficacy is reported to reduce parents’ experience of stress when they observe adolescents face challenges [40]. There is a need to explore possible associations between parental factors and adolescents’ ability to believe in their own capacity when facing challenges.

There is a lack of research evidence exploring the potential relationship between parental self-efficacy, HRQOL, stress and educational level on Norwegian adolescents’ self-efficacy. Hence, this paper sought to address the current research gaps by using the following aims:

1. To describe parental self-efficacy, HRQOL, stress, and educational level.
2. To explore associations between the participating parent’s self-efficacy, HRQOL, stress, and educational level on adolescents’ self-efficacy.

## Methods

### Design

This cross-sectional study was a part of the “Start Young—quality of life and pain in generations” study. The primary aim of this longitudinal study was to acquire new knowledge about HRQOL and pain in adolescents and their parents [26]. The current study utilized baseline data from November 2018 to April 2019. Baseline data was collected for the specific purposes in “Start Young”, including this current paper.

### Study setting

A total of 59 elementary schools from the south-eastern part of Norway were invited to participate, which potentially could have included 1663 adolescents from the 9th grade with one corresponding parent each. In total, 696 adolescents (41.8%) were included at baseline and 561 mothers or fathers filled out the questionnaire (33.7%). Herein, we included 508 adolescent-parent dyads (30.5% of the invited). Among the 508 participating parents, 393 were mothers and 115 were fathers. Of the 59 invited schools, 22 schools agreed to participate. These participating schools varied in size and localization (from city to suburb), indicating a variety of different sociocultural and economic backgrounds. The response rate varied across schools from 2.9% to 71.1%, but due to GDPR restrictions we were not allowed to follow-up the non-responders.

### Study procedures

One researcher visited each school approximately 1 week before the data collection to provide the adolescents with verbal and written information about the study. Written information was also distributed to the parents. Informed consent was obtained from both adolescents and their parents. A web-based test battery of questionnaires was administered and completed by adolescents in the classrooms during school hours. All schools provided the adolescents with computers to complete the questionnaire. One researcher and a teacher were present to aid when needed. Parents received a mail with a safe link to the test battery of questionnaires which they completed in their spare time. A safe data server was used to store the collected data [41]. We used information from the parents’ consent form to link the adolescents’ questionnaires with their parents’ questionnaires by creating a mutual ID number.

The “Start Young” study was reviewed by the Norwegian Centre for Research Data (Reference number: 60981), and the necessary approvals were obtained.

### Measures

#### Demographic variables

The baseline questionnaire administered to the participating adolescent-parent dyads and included questions regarding sociodemographic data such as gender and age. The participating parent also answered questions about their educational level, which was recoded into three categories: Low educational level = ≤ 12 years of education. Medium educational level = 13–15 years of education and high educational level = ≥ 16 years of education. Further, the baseline questionnaire of the “Start young” project included the general self-efficacy questionnaire for both adolescents and parents and the parents also answered the following questionnaires: stress and HRQOL.

#### Questionnaires

##### Self-efficacy

Self-efficacy was assessed by both adolescents and parents using the Norwegian 10-item version of the General Perceived Self-Efficacy Scale (GSE) [42]. GSE is developed to identify a person’s optimistic self-belief and global confidence in one’s abilities to cope with the tasks, demands and challenges of life in general, and is considered a valid and reliable psychometric scale, both in adolescence and adulthood [43–45]. The Cronbach’s alpha value is reported to be 0.87 among adolescents in this current study [26, 46]. GSE consists of 10 statements rated on a scale from 1 (completely wrong) to 4 (completely right). The individual GSE scores are then summed for a total score and divided by 10 to construct an average self-efficacy score. Higher GSE scores indicate higher levels of self-efficacy.

##### Health-related quality of life in parents

Health-related quality of life in parents was assessed using the 36-item Medical Outcomes Study Short Form (RAND-36). The instrument is comprised of eight domains (general health, bodily pain, physical function, physical role limitations, mental health, vitality, social function and emotional role limitations), with a Cronbach’s alpha value of 0.84 among this study sample [46, 47]. The domains are assembled into a physical component summary health scale (PCS) and a mental component summary health scale (MCS) [48, 49]. PCS and MCS scales were scored according to recommended scoring procedures, of which the items used values from 0 to 100 [48, 49]. Higher values indicate better HRQOL. The Norwegian RAND-36 is reported to be a valid and reliable tool for measuring HRQOL [50].

##### Stress

Stress in parents was assessed using the 30-item Perceived Stress Questionnaire (PSQ) which refers to the last four weeks and contains both negatively and positively formulated items that are rated on a 4-point rating scale [51]. Answers were recoded so that higher scores indicate higher levels of perceived stress. The resulting PSQ total score was transformed linearly between 0 and 1: PSQ = (raw value − 30)/90. Commonly used cutoff levels of stress within PSQ are low: < 0.33, medium: 0.33–0.45, moderate: 0.46–0.60 and severe: >0.60 [52]. The Norwegian version of PSQ has shown good validity and reliability [53], with a Cronbach’s alpha value of 0.93 within this study sample [26, 46].

### Statistical analyses

The descriptive analyses were conducted using IBM SPSS Statistics for Windows, Version 25.0 (IBM Corp., Armonk, NY, USA), wherein continuous variables were described using mean and standard deviation and categorical variables are presented as percentages.

Regression analyses were conducted using STATA software (Stata Statistical Software: Release 17. College Station, TX: StataCorp LLC.). Multivariable regressions were conducted between the predicting parental independent study variables (self-efficacy, HRQOL, stress and educational status) on the dependent variable (adolescents’ self-efficacy). Crude analyses with 95% confidence intervals were presented. Given sociodemographic variables such as educational level were included in the multivariable regression analyses, adjusting for SES was not considered appropriate. Adjusting for other possible confounders such as adolescents’ age was not considered applicable due to the homogeneity of the sample. Due to the fact that the web-based test battery of questionnaires included mandatory answering categories, no imputation of missing values was considered necessary. As the study is considered exploratory, no correction for multiple testing was done. For measuring the strength of the separate multivariable regressions for mothers and fathers, confidence intervals (CI) and standardized beta coefficients were presented. *p*-values < 0.05 were considered statistically significant and all tests were two-sided.

## Results

### Participants

This study included 508 school-based Norwegian adolescents and their corresponding mother or father. Adolescents’ mean age was 14.1 years (SD, 0.3) comprised of 55.3% girls and 44.7% boys with a mean self-efficacy score of 3.1 (SD, 0.4). Parent’s mean age was 45.3 years (SD, 4.9).

Descriptive data of parental self-efficacy, HRQOL, stress and educational level.

Descriptive analyses revealed a self-efficacy score of 3.3 (0.4) vs. 3.4 (0.4) (mean/SD), HRQOL (mean/SD, PCS 51.1 (9.5) vs. 53.3 (7.0), MCS 51.9 (8.2) vs. 54.1 (7.2)) and stress (mean/SD, 0.28 (0.24) vs. 0.25 (0.15) among mothers and fathers respectively (see Table 1). About half of the mothers (50.6%) and 46.1% of the fathers reported ≥ 16 years of education.

**Table 1:** Self-efficacy, stress, HRQOL and educational status of parents by gender

Study variable	Mothers (*n* = 393) (mean/SD)	Fathers (*n* = 115) (mean/SD)	Parents (*n* = 508) (mean/SD)
Self-efficacy	3.3 (0.4)	3.4 (0.4)	3.3 (0.4)
HRQOL			
PCS MCS	51.1 (9.5) 51.9 (8.2)	53.3 (7.0) 54.1 (7.2)	51.6 (9.0) 52.4 (8.0)
Stress^a^	0.28 (0.24)	0.25 (0.14)	0.27 (0.15)
Educational status N (%)^b^			
≤ 12 years of education	100 (26.5)	27 (23.5)	117 (25.0)
13–15 years of education	94 (23.9)	35 (30.4)	129 (25.4)
≥ 16 years of education	199 (50.6)	53 (46.1)	252 (49.6)

^a^*N* = 507 (missing data on one mother)

^b^Recoded into three categories: Low educational level = ≤ 12 years of education. Medium educational level = 13–15 years of education and high educational level = ≥ 16 years of education

### Associations between parental self-efficacy, HRQOL, stress and educational level on adolescents’ self-efficacy

Multivariable regressions of maternal study variables on adolescents’ self-efficacy revealed all nonsignificant relations (Table 2). An absence of evidence for an association was found between maternal self-efficacy and adolescents’ self-efficacy (95% CI [−0.13 to 0.12]).

**Table 2:** Multivariable regressions of maternal factors (independent variables) on adolescents’ self-efficacy (dependent variable)

Study variable	B	95% CI	*p*-value
Self-efficacy	−0.00	−0.13 to 0.12	0.95
HRQOL			
PCS MCS	−0.03 0.16	−0.08 to 0.02 −0.07 to 0.10	0.29 0.69
Stress	−2.96	−7.64 to 1.71	0.21
Educational status^			
≤ 12 years of education (ref)			
13–15 years of education ≥ 16 years of education	−0.52 0.52	−1.83 to 0.79 −0.65 to 1.69	0.44 0.38

^Recoded into three categories: Low educational level = ≤ 12 years of education. Medium educational level = 13–15 years of education and high educational level = ≥ 16 years of education

Multivariable regressions revealed all nonsignificant associations between paternal study variables on adolescents’ self-efficacy (Table 3).

**Table 3:** Multivariable regressions of paternal factors (independent variables) on adolescents’ self-efficacy (dependent variables)

Study variable	B	95% CI	*p*-value
Self-efficacy	0.02	−0.16 to 0.20	0.86
HRQOL			
PCS MCS	−0.02 −0.06	−0.13 to 0.08 −0.22 to 0.11	0.65 0.50
Stress	−2.71	−10.99 to 5.57	0.52
Educational status^			
≤ 12 years of education (ref)			
13–15 years of education ≥ 16 years of education	−1.70 −0.94	−3.78 to 0.37 −2.88 to 1.00	0.11 0.34

^Recoded into three categories: Low educational level = ≤ 12 years of education. Medium educational level = 13–15 years of education and high educational level = ≥ 16 years of education

## Discussions

In this study, we aimed to describe parental factors and to explore associations between the participating parent’s self-efficacy, HRQOL, stress, and educational level on adolescents’ self-efficacy stratified by gender.

Multivariable regressions revealed nonsignificant associations between the parental predictors variables and adolescents’ self-efficacy.

In this current study descriptive analyses revealed general self-efficacy mean scores of 3.3 and 3.4 out of 4, for mothers and fathers respectively. When aligning these findings to other countries, both German and American adults report lower average scores around 2.9 [54], indicating relatively high self-efficacy among the parents participating in this study. However, differences in the location of means between countries could be explained by the degree to which the sample is self-selected and other study characteristics. The German adult sample was reported as a heterogenous sample, while our study sample were more homogeneous because of the research design. Parental self-efficacy is reported to be a powerful direct predictor of positive parenting practices [55]. Hence, the parental self-efficacy seems to be one of several relevant characteristics throughout the childhood and presumably impacts the children’s developmental trajectory related to several aspect of life [56]. In a more recent systematic review examining the role of parental self-efficacy in parents and children’s well-being by Albanese et al., findings revealed that parental self-efficacy positively impacts child development and parent-child relationship [57]. However, in the transition from childhood to adolescence, several new aspects arise, such as identity formation, developmental changes, and influence from peers, which could together accumulate to a challenging period for the parents and/or caregivers. In early adolescence, higher level of independence is reported to be highly important for the adolescent and thus parents’ impacts are presumably reduced [1, 2].

Our descriptive findings revealed somewhat higher levels of HRQOL among fathers than mothers, however both groups reported scores above norm [58]. Moreover, half of the parents reporting 16 years of education or more, presumably indicating a relatively high socioeconomic group of parents and/or caregivers. However, a review of the literature by Hanson et al. that examined the relationship between socioeconomic status and health behaviors in adolescence found that these associations are weaker in adolescence compared to adulthood [59]. Hence, regardless of socioeconomic status, adolescents appear to be in a developmental phase characterized by risk-taking behaviors [60], and are influenced more by peers and immediate rewards than by parental guidance or consideration of long-term consequences [61].

The reported perceived stress scores among parents and/or caregivers aligns with stress levels categorized as low [51]. Earlier research evidence has suggested a relation between adolescence autonomy and parental stress, off which mothers stress levels was significantly related to their children’s desire for greater autonomy [62]. Even though these mechanisms may still be applicable, the embarkment of technology and social media the last years should not be neglected, as these platforms presumably make it more challenging for the parents to control external factors influencing adolescent’s belief, values, autonomy and self-efficacy. Still, the parental supportive communication is reported to be of importance when adolescents receiving ongoing video updates on a constant basis, as it seems to reduce the adolescents’ fear of missing out (FoMo) [63]. As social media seems nowadays to be an essential communication platform for family relationships, research evidence suggest the need to foster positive perceptions about social media’s potential impact on their family relationships [64]. Social media self-efficacy (SMSE) was conceptualized in 2014, which entails how people evaluate information found online based on their understanding of the their perceived ability to reach desired outcome [65].

From a statistical perspective, given the study sample comprised of over 3 times more mothers than fathers, the basis for stronger statistical strength for the mothers were also present. Still, nonsignificant associations were revealed. In light of the proposed theoretical components of Bandura [5] and the systematic review with meta-analysis by Ashford and colleagues [14], our findings highlight that the observations of others may presumably be more linked to peers than parents and/or caregivers in early adolescence. In addition, other components which have shown to be effective in increasing self-efficacy such as feedback by comparing performances and feedback on participants past performances may also be more linked to peers and adolescents’ environment, rather than the parental guidance. Bandura suggested verbal persuasion as an important component for increasing self-efficacy, however there is a lack of support for this approach in the literature [14]. Resistance to parental authority and verbal persuasion is likely a natural part of the process of establishing independence during adolescence. Despite that the parental study variables included in the current study did not reveal any associations on adolescents’ self-efficacy, there is large research evidence supporting the importance of parental self-efficacy on positive impacts of children wellbeing into early adolescence [55, 57]. However, parental self-efficacy is reported to decrease when the children enters early adolescence due to the quality of parent-adolescents communication, physical changes in the child and expectations for risk taking [66]. Hence, more research evidence is needed to identify factors influencing self-efficacy in early adolescence and grasp how parental self-efficacy and other factors are linked to adolescents’ self-efficacy for providing the most appropriate parental guidance.

### Strengths and limitations

There are several strengths that should be highlighted as the use of well-validated questionnaires, such as the general self-efficacy scale used by both parents and adolescents, which have shown consistent evidence of validity among different cultures and samples and is considered as a universal construct that yields meaningful relations with other instruments [67]. Moreover, it should be considered a strength that this study extends previous assumptions and research evidence by addressing a clear gap in the literature and pursue to publish nonsignificant findings of associations. On the other hand, several limitations should be considered, as the parents in the study exhibit very high self-efficacy scores with little variation, which reduces the scale’s ability to differentiate between individuals and limits its effectiveness in detecting meaningful associations with other variables. The utility of the instrument is limited in this case due to the highly self-selected sample and the cross-sectional study design provides a snapshot of the study sample and thus cannot identify any causal associations. Based on the multivariable statistical approach, the homogeneity in adolescents’ age and current nonsignificant findings, adjusting for additional covariates was not considered appropriate. Further, these findings may only be generalized to a specific population of Norwegian parents. Based on the available literature, we assumed differences in the strength of the associations between the predictor and outcome, rather than testing for relevant interactions, which should be considered a major limitation. Further, more comprehensive statistical analyses should be addressed to understand the complexity in the associations, as our simple current models has major limitations. Other explanations for the non-significant findings may be due to the design, or other measurement issues with the variables. Our simple regression models may not adequately capture the complex mechanisms linking parental factors to adolescent’s self-efficacy as these relations are likely influenced by unmeasured variables and dynamic indirect processes that are not accounted for. These factors should be taken into consideration when interpreting the findings. The decision regarding which parent participated may have been influenced by factors such as educational level or perceived parental competence, possibly leading to a non-representative sample of mothers and fathers. This self-selection may have attenuated the observed gender differences and limits the generalizability of the findings. Finally, the high number of nonparticipating schools should be considered as a limitation as the risk for selection bias increases. Due to GDPR we were not allowed to pursue these nonparticipating schools. Future research could benefit from more comprehensive models incorporating mediators, moderators and other constructs to get an increased insight in this complex picture.

## Conclusions

In this study, the parental factors examined were not associated with adolescents’ self-efficacy. These findings highlight the need for further observational and longitudinal studies with larger samples and more comprehensive models to better understand how parental factors influence adolescents’ self-efficacy.

## Data Availability

The datasets used and/or analyzed during the present study are not publicly available due to the General Data Protection Regulation laws but are available from the corresponding author on reasonable request and with permission from the Norwegian Centre for Research Data.

## Abbreviations

CI: Confidence interval
GSE: General self-efficacy
PSQ: Perceived stress questionnaire
RAND-36: 36-item medical outcomes study short form
PSC: Physical component summary health scale
MCS: Mental component summary health
SD: Standard deviation
HRQOL: Health-related quality of life
FOMO: Fear of missing out
SMSE: Social media self-efficacy
